# Multidisciplinary clinic model enhances liver and metabolic health outcomes in adults with MASH

**DOI:** 10.1097/HC9.0000000000000649

**Published:** 2025-02-03

**Authors:** Jonathan G. Stine, David Bradley, Jennifer McCall-Hosenfeld, Victoria Motz-Patel, Justin Tondt, Sarah Batra, Britney Fitzgerald, Samantha Garcia, Breianna Hummer, Courtney Kindrew, Autumn Koppenhaver, Hannah Mohr, Nataliya Smith, Heather Tressler, Kyra VanKirk, Karen Krok, Ian Schreibman, Elizabeth Stonesifer, Kofi Clarke

**Affiliations:** 1 Department of Medicine, Division of Gastroenterology and Hepatology, Penn State Health Milton S. Hershey Medical Center, Hershey, Pennsylvania, USA; 2 Fatty Liver Program, Penn State Health Milton S. Hershey Medical Center, Hershey, Pennsylvania, USA; 3 Liver Center, Penn State Health Milton S. Hershey Medical Center, Hershey, Pennsylvania, USA; 4 Department of Public Health Sciences, The Pennsylvania State University, College of Medicine, Hershey, Pennsylvania, USA; 5 Cancer Institute, Penn State Health Milton S. Hershey Medical Center, Hershey, Pennsylvania, USA; 6 Department of Medicine, Division of Endocrinology, Penn State Health Milton S. Hershey Medical Center, Hershey, Pennsylvania, USA; 7 Department of Medicine, Penn State Health Milton S. Hershey Medical Center, Hershey, Pennsylvania, USA; 8 Department of Family Medicine, Penn State Health Milton S. Hershey Medical Center, Hershey, Pennsylvania, USA; 9 College of Medicine, Penn State University, Hershey, Pennsylvania, USA

**Keywords:** fatty liver, metabolic dysfunction–associated steatotic liver disease, NAFLD

## Abstract

**Figure:**
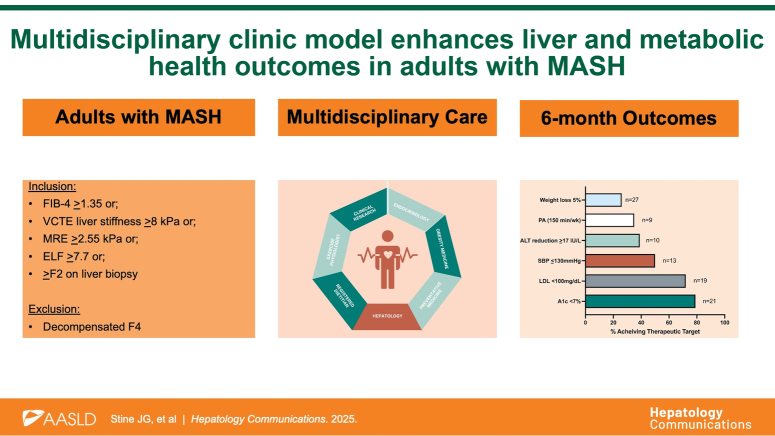

## INTRODUCTION

Because metabolic dysfunction–associated steatotic liver disease (MASLD) exists within the context of greater multisystem organ dysfunction, a broad approach is necessary to improve outcomes for the billions with MASLD.^1^ Given treating metabolic dysfunction in silos is ineffective, growing interest in multidisciplinary care models has emerged and leading hepatology societies endorse a multidisciplinary approach.^2^,^3^ Despite this guidance, multidisciplinary care is not widespread; only 30% of US academic medical centers offer this.^4^ Moreover, there is no agreed-upon care model that consistently improves outcomes, programs are rarely co-located in the same clinic, and important specialists (eg, obesity medicine and exercise physiologists) are missing from most programs.^4^,^5^,^6^ Recognizing the unmet clinical need for comprehensive, multidisciplinary care, we designed and implemented a large program of seven specialists that are co-located in the same clinical space to offer a single stop for the patient. We hypothesized that this expansive multidisciplinary program would improve patient outcomes at clinically significant thresholds of response. This paper details our experience.

## METHODS

**FIGURE 1:**
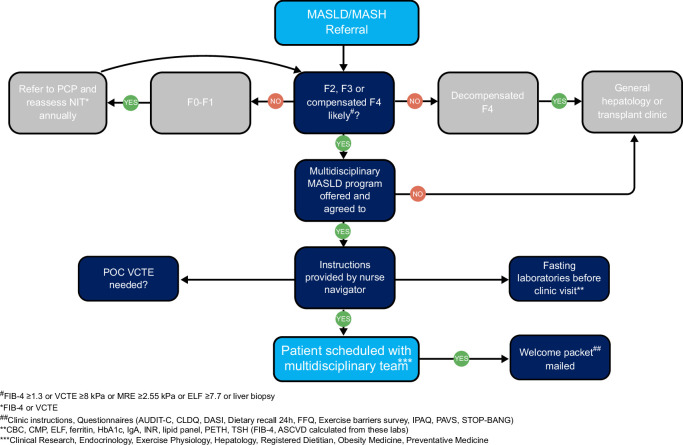
Multidisciplinary MASLD clinic referral pathway and preclinic visit testing. Abbreviation: AUDIT-C, Alcohol Use Disorders Identification Test; ASCVD, atherosclerotic cardiovascular disease; CBC, complete blood count; CLDQ, Chronic Liver Disease Questionnaire; CMP, complete metabolic panel; DASI, Duke Activity Status Index; ELF, Enhanced Liver Fibrosis; F, fibrosis; FFQ, Food Frequency Questionnaire; FIB-4, Fibrosis-4 Index; HbA1c, hemoglobin A1c; IgA, immunoglobulin A; IPAQ, International Physical Activity Questionnaire; INR, international normalized ratio; MASLD, metabolic dysfunction–associated steatotic liver disease; MRE, magnetic resonance imaging elastography; NIT, noninvasive test; PAVS, Physical Activity Vital Sign; PETH, phosphatidylethanol; POC, point of care; PCP, primary care provider; TSH, thyroid stimulating hormone; VCTE, vibration-controlled transient elastography.

Figure 1 summarizes the program structure, referral pathway, pre-visit counseling, and required testing. On the day of the clinic visit, the patient proceeds through sequential appointments co-located in the same clinic, during which time questionnaires, laboratories, and imaging tests are reviewed. Each patient receives standardized lifestyle counseling regarding (1) consuming a hypocaloric Mediterranean-informed diet with a goal of 10% weight loss^7^; (2) performing guideline-based physical activity^8^; (3) consuming three 8 oz cups of coffee per day and avoiding sugar-sweetened beverages; (4) sleeping ≥7 hours per night; (5) avoiding excessive alcohol; (6) smoking cessation. Personalized meal plans and exercise prescriptions are provided where appropriate.

Patients are also counseled on the benefits of drug therapy for metabolic dysfunction–associated steatohepatitis and/or metabolic comorbidities. Systolic blood pressure (SBP) <130 mm Hg, LDL <100 mg/dL, and hemoglobin A1c <7% are targeted. If the patient meets established criteria for antiobesity medications or clinical trials, these are offered. Referrals are also made to bariatric surgeons, endobariatricians, sleep medicine experts, and a clinical psychologist, each of whom interacts regularly with the program, as needed. Once the initial consultation is complete, clinical data and individual provider recommendations are reviewed at the biweekly multidisciplinary MASLD board; treatment plans are adjusted as appropriate and communicated to the patient.

Patients follow up in person 1 year later to complete the same procedures as their initial consultation. Prior to that, they are contacted 6 months after their initial consultation by phone. An interim history is obtained, including assessment for changes in body weight, physical activity, medication compliance, and medication side effects. The information is routed to individual members of the treatment team as appropriate. Additionally, the patient is asked to complete interim laboratory tests (eg, complete blood count, complete metabolic panel, ferritin, hemoglobin A1c, IgA, and lipid panel), and adjustments to the treatment plan are made.

### Data analysis plan

The statistical analysis in this paper compares changes between the initial consultation and the information obtained at 6 months. Continuous variables are analyzed with paired *t* tests, and categorical variables with a chi-square test where appropriate. A *p*-value of <0.05 is statistically significant. All analyses were conducted with SAS version 9.4 (Cary, NC).

## RESULTS

*Overall program registry characteristics.* Seventy-eight consecutive adults were enrolled between October 2023 and August 2024. Salient features are summarized in Supplemental Table S1, http://links.lww.com/HC9/B890. Briefly, the cohort was largely non-Hispanic Whites (85%) with F2/F3 (80%) disease and multiple metabolic comorbidities (67% hypertension, 50% hyperlipidemia, and 42% diabetes).

*Impact on liver and metabolic health.* For the twenty-six patients who had data available at 6 months (Supplemental Table S1, http://links.lww.com/HC9/B890), several improvements were noted (Supplemental Figure S1, http://links.lww.com/HC9/B890), both for liver health, including 39% (n=10) achieved clinically meaningful reduction in ALT,^9^ and metabolic health: 79% of patients (n=21) achieved A1c <7%, 72% LDL <100 mg/dL (69% of patients with diabetes), 50% SBP ≤130 mm Hg, 35% achieved guideline-based amounts of weekly physical activity and 26% body weight loss of ≥5%.

## DISCUSSION

This study demonstrates that our multidisciplinary care model for adults with metabolic dysfunction–associated steatohepatitis and significant liver fibrosis improves multiple outcomes and at clinically meaningful levels of response, illustrating the important benefits of a comprehensive care team approach. The program achieved this through individualized treatment plans, which included a combination of patient education, lifestyle intervention, and drug therapy, which were agreed upon after individual evaluation and multidisciplinary team discussion, with the goal of achieving preagreed-upon therapeutic targets. This is important because while multidisciplinary care models have been proposed and endorsed by several clinical practice guidelines, there is limited data on both efficacy and sustainability. Additionally, our program focuses on health care specialists that have been traditionally overlooked by other multidisciplinary care models—staff exercise physiologist, obesity medicine provider, and a clinical psychologist available for referral. Adding these essential specialists to our team was a key element in the program’s efficacy.

Potential limitations of this study include a short follow-up period that did not capture liver-related events, no comparator arm, small sample size, and potential for selection bias. Strengths are numerous and include a comprehensive multidisciplinary approach with a wide range of specialists, observed efficacy across multiple clinically significant therapeutic targets, and real-world implementation in a robust population. Further studies are necessary to not only validate our findings but also to evaluate the cost-effectiveness of multidisciplinary care models. In addition, this care model needs to be validated in a more diverse population. Exploring these future directions will allow us to better refine multidisciplinary care models for adults with MASLD, and to make them widely accessible in a cost-effective and adaptable manner.

## CONCLUSIONS

Although multidisciplinary care models are endorsed by leading clinical practice guidelines to help address the many complexities unique to clinical management of MASLD, limited evidence remains to inform on their effectiveness in a real-world setting. This study provides novel evidence that a comprehensive, multidisciplinary care model involving a wide range of specialists can lead to significant improvement in liver and metabolic health through sustained lifestyle intervention in conjunction with targeted pharmacologic therapies. These findings suggest that expanding multidisciplinary care programs could greatly improve clinical outcomes for all patients with MASLD.

## Supplementary Material

SUPPLEMENTARY MATERIAL
