# Supplementary material for: Multidisciplinary clinic model enhances liver and metabolic health outcomes in adults with MASH
Source: Hepatol Commun. 2025 Feb 3;9(2):e0649. doi: 10.1097/HC9.0000000000000649 (PMC12333794; doi:10.1097/HC9.0000000000000649)
Supplement: SUPPLEMENTARY MATERIAL [file hc9-9-e0649-s001.docx]

**Table S1. Baseline and 6-month follow-up data for patients enrolled in the MASLD multidisciplinary care program**

|  | **Entire Cohort Baseline (n=78)** | **Follow-up Cohort Baseline (n=26)** | **Follow-up Cohort 6 Month Visit (n=26)** |
| --- | --- | --- | --- |
| **Age, years** | **57.0 (47.3-65.8)** | **61.0 (49.0-69.0)** | **-** |
| **Female sex, n (%)** | **42 (54)** | **16 (57)** | **-** |
| **Weight, kg** | **101.1 (90.9-118.8)** | **96.6 (88.6-111)** | **92.2 (84.1-106.1)** |
| **BMI, kg/m^2^** | **34.3 (31.1-39.0)** | **33.1 (30.2-37.1)** | **31.6 (29.9-36.7)** |
| **Race/ethnicity, n (%)**  **White**  **Hispanic**  **Asian** | **68 (87)**  **5 (6)**  **5 (6)** | **24 (92)**  **2 (8)**  **2 (8)** | **-**  **-**  **-** |
| **Metabolic comorbidities, n (%)**  **Hypertension**  **Hyperlipidemia**  **Diabetes** | **52 (67)**  **39 (50)**  **33 (42)** | **21 (81)**  **18 (69)**  **13 (46)** | **-**  **-**  **-** |
| **Physical activity (min/wk.)** | **60 (0-180)** | **60 (0-140)** | **90 (15-180)** |
| **MASH NITs**  **VCTE (kPa)**  **ELF** | **9.9 (7.4-13.4)**  **9.7 (9.2-10.0)** | **8.5 (6.8-10.8)**  **9.7 (9.2-10.0)** | **-**  **-** |
| **Liver fibrosis stage (2/3/4)** | **42 (54)/ 20 (26)/ 16 (20)** | **17 (65) / 7 (27) / 2 (8)** | **-** |
| **Liver enzymes, IU/L**  **AST**  **ALT** | **33.0 (24.0-47.0)**  **40.5 (28-59.3)** | **34.5 (26.0-44.8)**  **48.0 (35.3-67.0)** | **23.5 (19.8-29.5)**  **32.5 (24.8-40.8)** |
| **Lipids, mg/dL**  **Total cholesterol**  **LDL**  **HDL**  **Triglycerides** | **170 (163-202)**  **99 (75-124)**  **41 (37-49)**  **142 (100-213)** | **164 (146-201)**  **86 (66-121)**  **40 (36-48)**  **151 (111-225)** | **143 (127-167)**  **76 (62-95)**  **39 (35-48)**  **123 (88-176)** |
| **A1c, %** | **6.1 (5.4-7.1)** | **6.1 (5.5-7.6)** | **5.9 (5.3-6.3)** |
| **Blood pressure, mmHg**  **SBP**  **DBP** | **130 (121-143)**  **81.5 (78-87)** | **131 (124-144)**  **81 (78-86)** | **127 (122-141)**  **81 (77-83)** |
| **10-yr ASCVD risk, %** | **9.1 (2.5-21.1)** | **5.8 (2.6-11.6)** | **3.6 (3.0-25.2)** |

**Continuous variables presented as median (IQR)**

**ALT=alanine aminotransferase; ASCVD=atherosclerotic cardiovascular disease; AST=aspartate aminotransferase; BMI=body mass index; DBP=diastolic blood pressure; ELF=enhanced liver fibrosis; HDL=high density lipoprotein; IU=international unit; LDL=low density lipoprotein; NIT=non-invasive test; SBP=systolic blood pressure; VCTE=vibration controlled transient elastography**

**Figure S1. Therapeutic targets were achieved frequently following 6 months of multidisciplinary care**

**
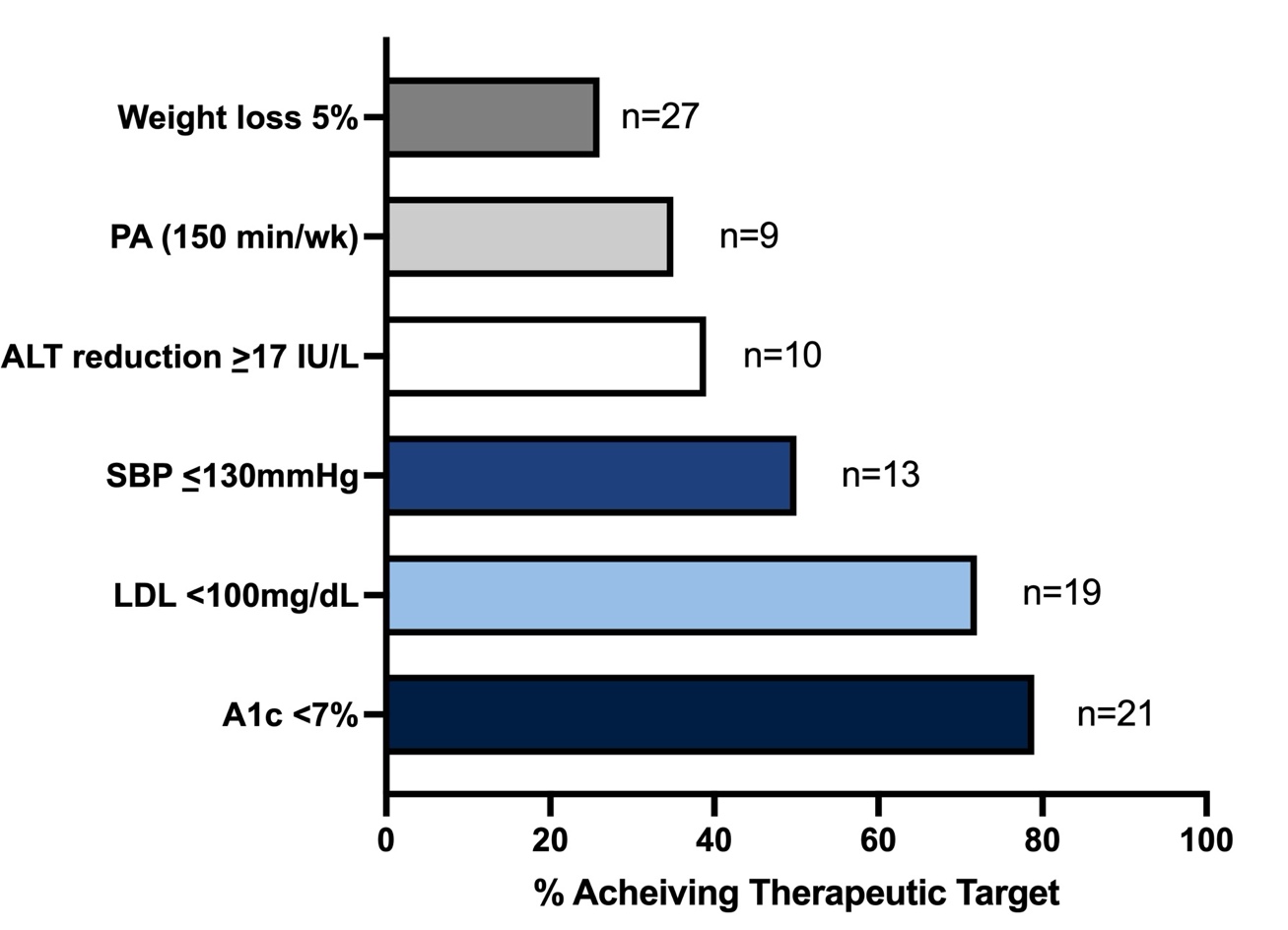
**
